# A novel reusable anti-COVID-19 transparent face respirator with optimized airflow

**DOI:** 10.1007/s42242-020-00097-1

**Published:** 2020-09-27

**Authors:** Hussain Alenezi, Muhammet Emin Cam, Mohan Edirisinghe

**Affiliations:** 1 Department of Mechanical Engineering, University College London, Torrington Place, London, WC1E 7JE UK; 2 Department of Manufacturing Engineering, College of Technological Studies, PAAET, 13092 Kuwait City, Kuwait; 3 Center for Nanotechnology and Biomaterials Application and Research, Marmara University, 34722 Istanbul, Turkey

**Keywords:** COVID-19, Mask, Prototype, Flow, Pressure, Temperature

## Abstract

This novel face mask is designed to be a reusable respirator with a small and highly efficient disposable fabric filter. Respirator material requirements are reduced by 75% compared to traditional designs and allow repeated cleaning or sterilization. The probability of virus particle inhalation is reduced using novel air filtration pathways, through square-waveform design to increase filter airflow. Air enters the mask from right and left side filters, while the area in front of the mouth is isolated. Clear epoxy is used for a transparent frame, allowing lip-reading, and mask edges contain a silicone seal preventing bypass of the filters. The mask is manufactured using silicone molds, eliminating electricity requirements making it economical and viable in developing countries. Computational fluid dynamics numerical studies and Fluent ANSYS software were used to simulate airflow through the filter to optimize filter air path geometry and validate mask design with realistic human requirements. The breathing cycle was represented as a transient function, and N95 filter specifications were selected as a porous medium. The novel design achieved 1.2 × 10^−3^ kg s^−1^, 20% higher than human requirements, with air streamlines velocity indicating local high speed, forcing and trapping virus particles against filter walls through centrifugal forces.

## Introduction

A significant threat to people’s lives lies in the spread of the coronavirus (entitled the COVID-19 pandemic) [1, 2]. Worldwide, 500,000 deaths have taken place, while COVID-19 confirmed cases are over 11 million according to the John Hopkins University Map and Dashboard [3]. COVID-19 has caused the demand for medical respirators to increase dramatically over the past 6 months, by at least sixfold [4–6].

World Health Organization (WHO) noted that the high demand compared to the usual trades volume for purchasing respirators led to a severe shortage in available quantity, which caused a price surge leaving frontline workers and patients in danger. This new pandemic calls for innovative and effective solutions requiring strong cooperation between medical, engineering science and industry experts [7–9].

The increased needs for personal protective equipment are based on mainly face masks, which play a crucial role in reducing virus transmission from one person to another as it is always difficult to control social distancing at all times. Globally, wearing a face mask in public areas is strongly supported, although there is a significant shortage. This shortage motivates many enthusiastic researchers to find new techniques for designing and manufacturing cheap but effective face masks with limited resources [10–12].

Face masks may be categorized into three groups, cloth, surgical, and N95 [13]. Due to the lack of face masks, many global health authorities recommended wearing homemade ones or those provided by any supplier. Generally, these are made by one cloth layer to prevent an infected person from transmitting their viruses to others by respiratory droplets [14]. Nevertheless, it has very low efficacy in drastically limiting the transport of viruses to the nose or mouth of the wearer. Moreover, the design consists of three layers manufactured by melt-blown polypropylene (PP) placed between the non-woven fabric of the surgical mask or fluid-resistant mask. The use of these masks during the pandemic was intensely debated [15]. A surgical mask is designed to help preventing large-scale droplets, sprays, and splashes rather than inhalation airborne of fine particles of viruses from entering the nose and mouth of the wearer; as a result, it is not certified as a respirator [16]. In contrast, N95 face-piece air filtration respirators are designed as elliptical or circular shaped to provide a secure fit to limit leakage of air on the sides. These masks deliver 95% filtration efficiency for airborne particles (~ 300 nm) and 85% for even finer ones throughout the pore size filter media if worn correctly [17–19]. As a result of shortage of these masks during the pandemic, some have been recommended that these masks only used by frontline healthcare workers. However, the wearers faced difficulties in breathing due to lower amounts of oxygen inhalation when used over a long time; therefore, different designs provided cumbersome exhalation valves [20].

During the COVID-19 pandemic, innovative mask designs emerged from around the globe, focusing on developing a disposable and reusable face mask. Reusable 3D printed face masks were designed and manufactured using different materials by several researchers [21]. Others modified the cloth mask by using five pairs of triboseries fabrics to increase the filtration efficiency [22], and the effect of heat and UV disinfection methods to sterilize N95 face masks was also studied [23].

Computational fluid dynamics (CFD) flow simulation was used to study the leakage in the face respirators in several case studies. For example, Lei et al. [24] studied a method for the inner flow within the volume formed between the mask and the headform surface, in addition to the flow outside of the mask using CFD and infrared imaging [24]. Others studied facial features and the impact of the flow region close to a breathing person’s nose or mouth [25].

In this unique and distinctly different research, a novel reusable face mask with square-waveform design is proposed. Product development to achieve optimized design is discussed. Additive manufacturing using 3D printing to customize the design to build silicone mold dies for cold molding of the masks is described. The geometry modification effect on the efficiency performance of the mask with velocity, pressure, and temperature distribution inside, across, and outside the mask is elucidated using flow simulation.

## Materials and methods

### Model design and development

In March 2020 due to various severe deficiencies in the manufacture of face masks, most factories had halted, and the availability of such masks had become limited in markets around the world. An efficient and cost-effective design was required to find a quick solution, and it was necessary to mass produce theses masks without electrical sources, so that this work can be adopted easily and economically adapted in developing countries too.

In the initial design, critical requirements must be met. For example, the mask needs to be manufactured without the availability of specialized personnel, it can be used several times after sterilization, and the filtration fabric can be replaced by any type that is available without the need for special tools. However, it is vital for the face piece of the mask to be leak-proof and prevent the entry or exit of air except through the filter. There must be enough space between the nostrils, mouth, and front of the mask to prevent deficiencies in the amount of oxygen inside the mask. Finally, the mask should be transparent to allow lip-reading, especially for the benefit of deaf people who suffer communication problems using non-transparent masks during the pandemic.

**Fig. 1:**
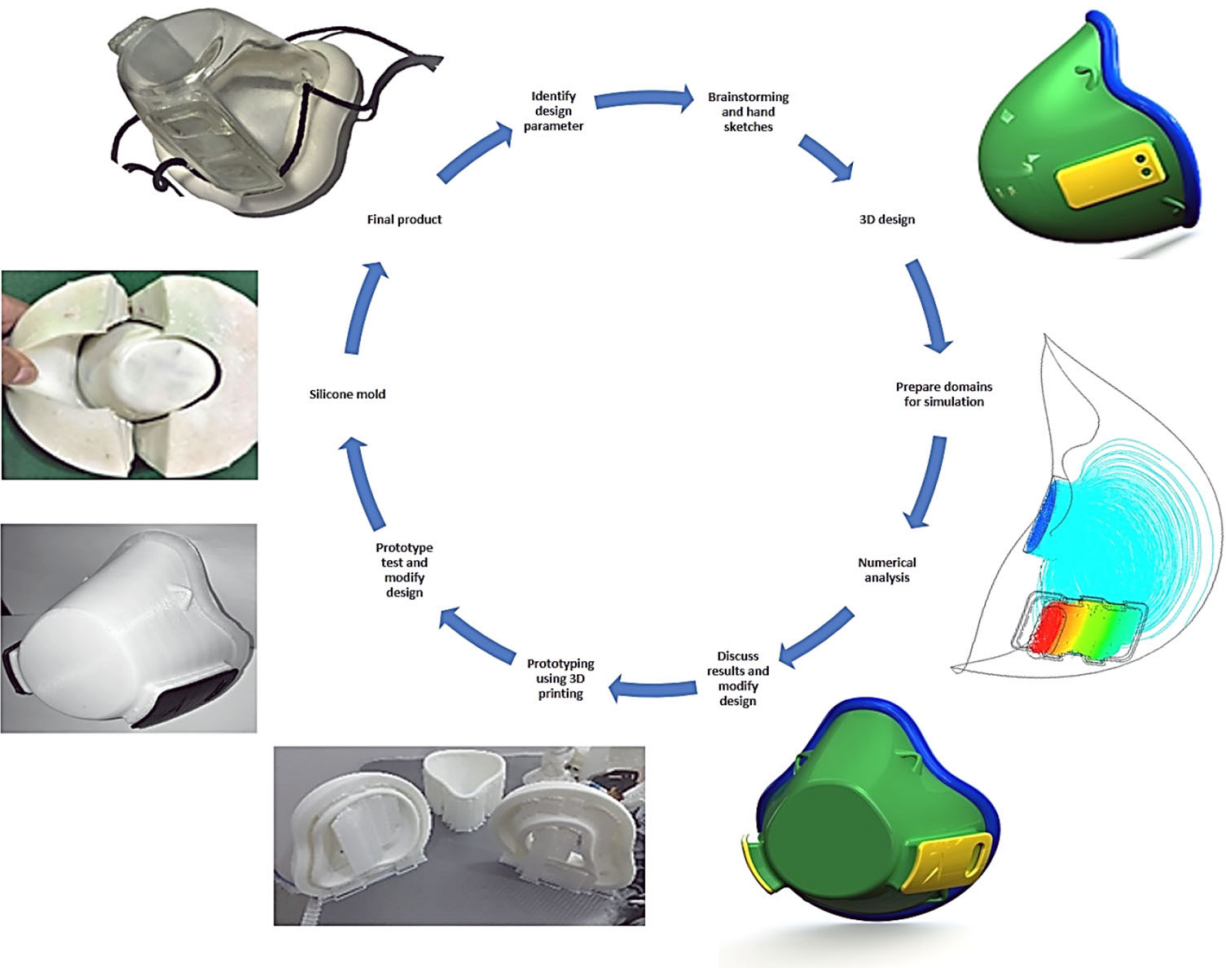
Process flowchart for the initial design and product development procedure using CAD, flow simulation, 3D printing, and cold molding processes

The process flowchart in Fig. 1 indicates the product development procedure, starting with hand sketches to identify the essential concepts. It was then converted to a 3D model using the SolidWorks^®^ CAD package to create suitable dimensions equivalent to the physical geometry of the real human face. Then, to examine the mask design, the preparation of the model domains to be used in the flow simulation was decided. Numerical simulation was used to analyze the amount of airflow, velocity, and pressure passing the filter medium and inside the mask, including temperature distribution in the face during breathing cycles, to ensure the design is meeting the requirements and was comfortable to the user. Design modifications were carried out to fulfill these requirements for the best suitable model.

### Novel design and prototyping features

The novelty in the proposed design is the way the filter works; the air tunnels are shaped like a square wave so that turbulent flow occurs. As a result, the air vortex causes the air to pass through the filter many times; this method adds two significant advantages: the first prevents particles from passing through the respiratory system, and the second is that the airborne particles and viruses are forced to spin at the filter area instead of moving with air to the breathing of the wearer.

**Fig. 2:**
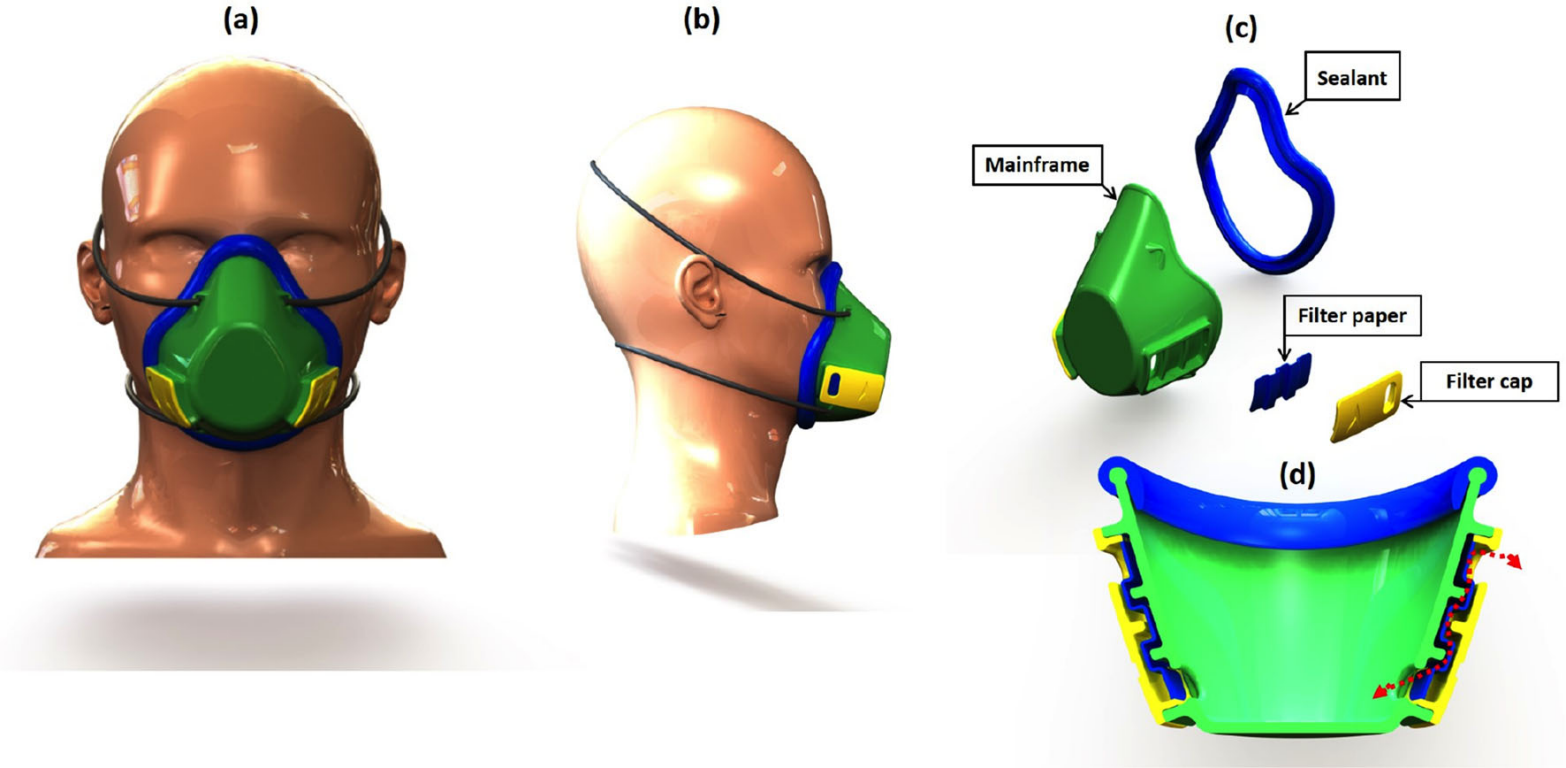
Photograph extracted from SolidWorks. **a** front, **b** side view of the final mask design, **c** disassembled mask parts, and **d** sectional front view of the filter area of the mask

As shown in Fig. 2d, the mask consists of four main parts: mainframe, two filter caps, filter material, and silicon sealant. The proposed filter area caused a reduction in filter consumption, which leads to the reduction of filter material required while maintaining the same efficiency, to solve the shortage caused by the pandemic, and to achieve material reduction of 75%. The user can form the filter manually with small pieces of textile, paper, etc., having 50 × 70 mm in dimension, simply by removing the filter caps, then sterilize the mask, and install new filters.

Prototyping is a vital step in product design, as shown in Fig. 1, to confirm the concepts. In this research, additive manufacturing 3D printing was used for prototyping, several models were made during the process, and modifying and reprinting were repeated to design a mask suitable for the face and capable of achieving the airflow required.

The material used for the mainframe and filter caps, as shown in Fig. 2d, is clear epoxy resin; this material is classified as safe; also, it is transparent to allow others to read the lips of users. This option provides added value of communication without more cost. Sealant made from silicone material is not toxic. The mask can be manufactured in different sizes with personalized fit.

Silicone molding was used to manufacture the masks [26]. The main feature in this process is that it does not require a power source like injection molding. Also, the process offers low initial cost, so it is also suitable for developing countries.

### Numerical model

The simulation model of airflow passing the filter medium and around the face was developed with the help of the CFD code (ANSYS-Fluent 19), to study the pressure, the velocity, and the temperature distribution for normal, and hard (deep) breathing. The scenario in between (middle) was also considered. The first step was to create a three-dimensional (3D) geometric model using the SolidWorks package and to generate an Ansys-fluent mesh by defining the setup method and boundary conditions. An unsteady, pressure-based transient-state solver was used for this analysis. The velocity during the breathing cycle has a function that has been created, and then user-defined functions (UDFs) were used to indicate the speed at the inlet of the nostrils.

### Physical geometry

**Fig. 3:**
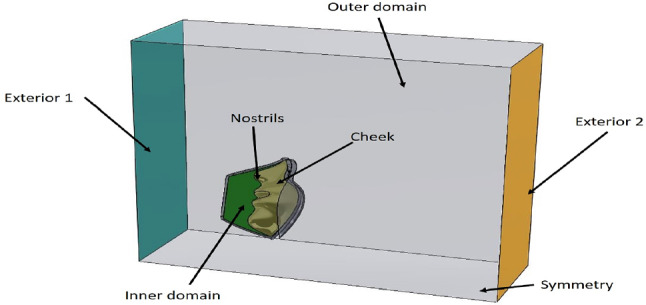
Three-dimensional model domains of this mask study

Figure 3 shows the physical structure of the 3D model of the mask used to determine the domains performed in the simulation. The air inlet surface is denoted by (Exterior 1), while the outer region is ambient air. The internal domain displays air from the inner surface of the mask to the cheek showing the source of heat. Besides, the filter domain is the volume between the inner and the outer regions. The nostrils indicate where breathing occurred, and other surfaces represent the external pressure as atmospheric pressure.

### Mesh generation

**Fig. 4:**
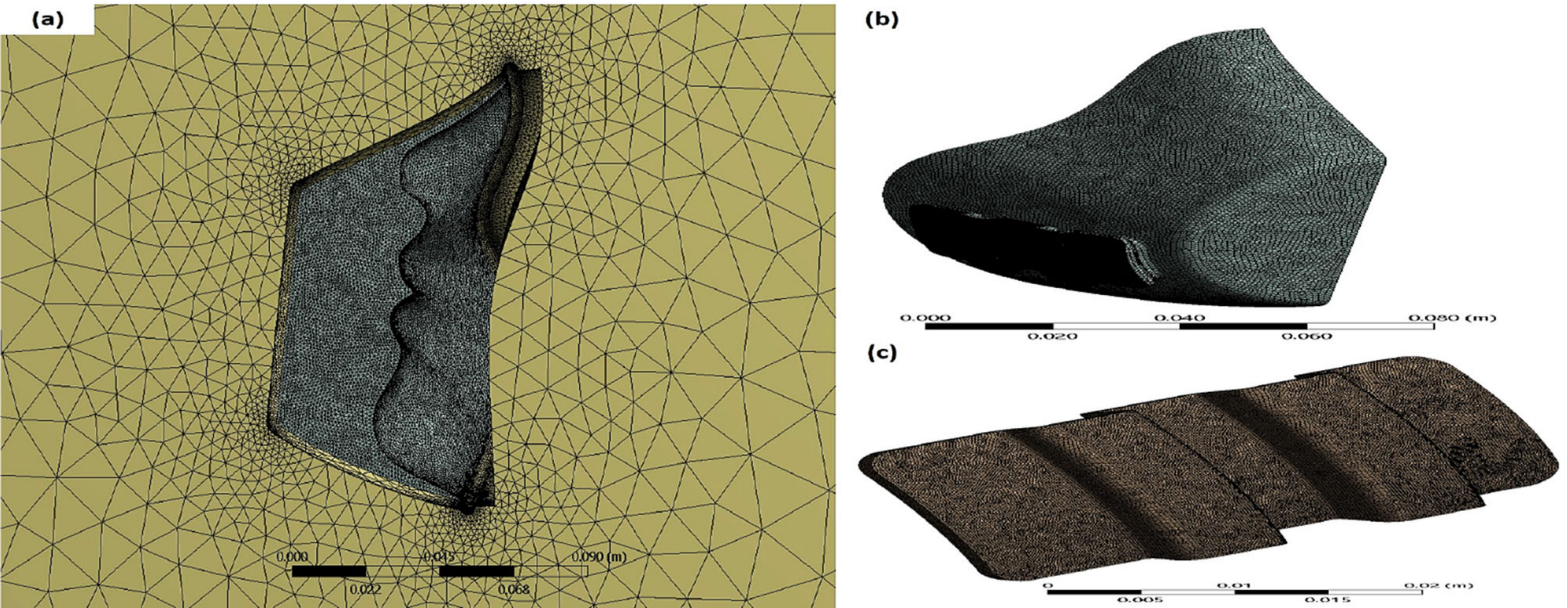
Mesh generation for **a** the full domain, **b** the inner domain, and **c** the filter domain

The unstructured tetrahedral grids, as shown in Fig. 4, were used for the computational domain with three grids of 265,487, 374,125, and 425,641 cells, respectively. There was no difference in results between the second and third configurations, so 425,641 cells were chosen. The inner domain and filter domain element sizes were selected as 1.0 and 0.25 mm, respectively. For the outer domain, auto-generation was used to reduce the number of elements.

### Governing equations

The conversion of the mass of air gas (g) and solid particles (viruses) that represent the continuity equation is given by [27, 28]:

1 $$ \frac{\partial }{\partial t}\left( {\varepsilon_{g} \rho_{g} } \right) + \nabla . \left( {\varepsilon_{g} \rho_{g} v_{g} } \right) = 0, $$

2 $$ \frac{\partial }{\partial t}\left( {\varepsilon_{s} \rho_{s} } \right) + \nabla . \left( {\varepsilon_{s} \rho_{s} v_{s} } \right) = 0. $$

The momentum equations of air gas (g) and solid particles (viruses) are determined by

3 $$ \frac{\partial }{\partial t}\left( {\varepsilon_{g} \rho_{g} v_{g} } \right) + \nabla . \left( {\varepsilon_{g} \rho_{g} v_{g} v_{g} } \right) = - \varepsilon_{g} \nabla p + \nabla .\tau_{g} + \varepsilon_{g} \rho_{g} g - \mathop \sum \limits_{s = 1}^{n} \beta_{gs} \left( {v_{g} - v_{s} } \right), $$

4 $$ \frac{\partial }{\partial t}\left( {\varepsilon_{s} \rho_{s} v_{s} } \right) + \nabla . \left( {\varepsilon_{s} \rho_{s} v_{s} v_{s} } \right) = - \varepsilon_{s} \nabla p + \nabla p_{s} + \nabla .\tau_{s} + \varepsilon_{s} \rho_{s} g - \mathop \sum \limits_{s = 1}^{n} \beta_{gs} \left( {v_{g} - v_{s} } \right) + F_{qs\alpha }. $$

Air (gas) and solid (virus) phases are represented by g and s, respectively, in which $v_{s}$ and $v_{g}$ are velocity vectors of the solid and gas phases, while $\beta_{gs}$ and $F_{qs\alpha }$ represent drag force and electrostatic force, respectively. The term p is the pressure, and $\rho$ and $\varepsilon$ are the density and volume fraction, respectively. Finally, *τ* is the stress tensor, which is given by:

5 $$ \tau = \mu \left[ {(\nabla \overset{\lower0.5em\hbox{$\smash{\scriptscriptstyle\rightharpoonup}$}} {v} + \nabla \overset{\lower0.5em\hbox{$\smash{\scriptscriptstyle\rightharpoonup}$}} {v}^{t} } ) - \frac{2}{3}\nabla .\overset{\lower0.5em\hbox{$\smash{\scriptscriptstyle\rightharpoonup}$}} {v}  I\right], $$

where the terms $\mu$, $I$, and *t* are dynamic viscosity, unit tensor, and time, respectively. The equations that present the velocity at the inlet of the nostrils for the three conditions are as follows:

Normal:

6 $$ {\text{v}} = 0.673t^{3} - 4.9557  t^{2} + 8.242   {\text{t}} - 0.2621; $$

Middle:

7 $$ {\text{v}} = 1.3461t^{3} - 9.9114  t^{2} + 16.485   {\text{t}} - 0.5243; $$

Deep:

8 $$ {\text{v}} = 4.0384  t^{3} - 29.734  t^{2} + 49.456   {\text{t}} - 1.5728. $$

### Boundary conditions

In this study, the breathing cycle for an adult was used to study the changes in pressure, volume flow rate, and temperature distribution during a single breathing cycle. During inhalation, the pressure is less than the atmospheric pressure, while during exhalation, it is higher than the atmospheric pressure. The boundary conditions used in this research are given in Table 1.

**Table 1:** Boundary conditions at various locations

Location	Boundary condition
Inlet of nostril	Velocity inlet: profiles of velocity were generated by UDF in the normal direction with temperature = 306 K
Exterior 1	Velocity inlet: velocity has a constant value = 1 ms^−1^ with normal direction condition at temperature = 300 K
Exterior 2	Pressure outlet: gauge pressure 0 Pa and temperature 300 K
Symmetry	The surface where the body is divided into two symmetric bodies
Cheek	Wall: initial temperature = 306 K
Other surfaces	Pressure outlet: gauge pressure 0 Pa and temperature 300 K
Outer domain	Atmospheric condition
Filter domain	Porous media, viscous resistance = 1.12e + 10 m^−2^ [29].
Virus simulation	Discrete phase, interaction with continuous phase, 100 nm diameter with temperature = 300 K

## Results and discussion

In this section, the effect of breathing levels (normal, middle and deep) on the volume flow rates and pressure was studied. Velocity, pressure, and temperature distribution were examined outside and inside the mask, the inner domain, and through the filter medium.

### Effect of breathing on mass flow and pressure in the mask

**Fig. 5:**
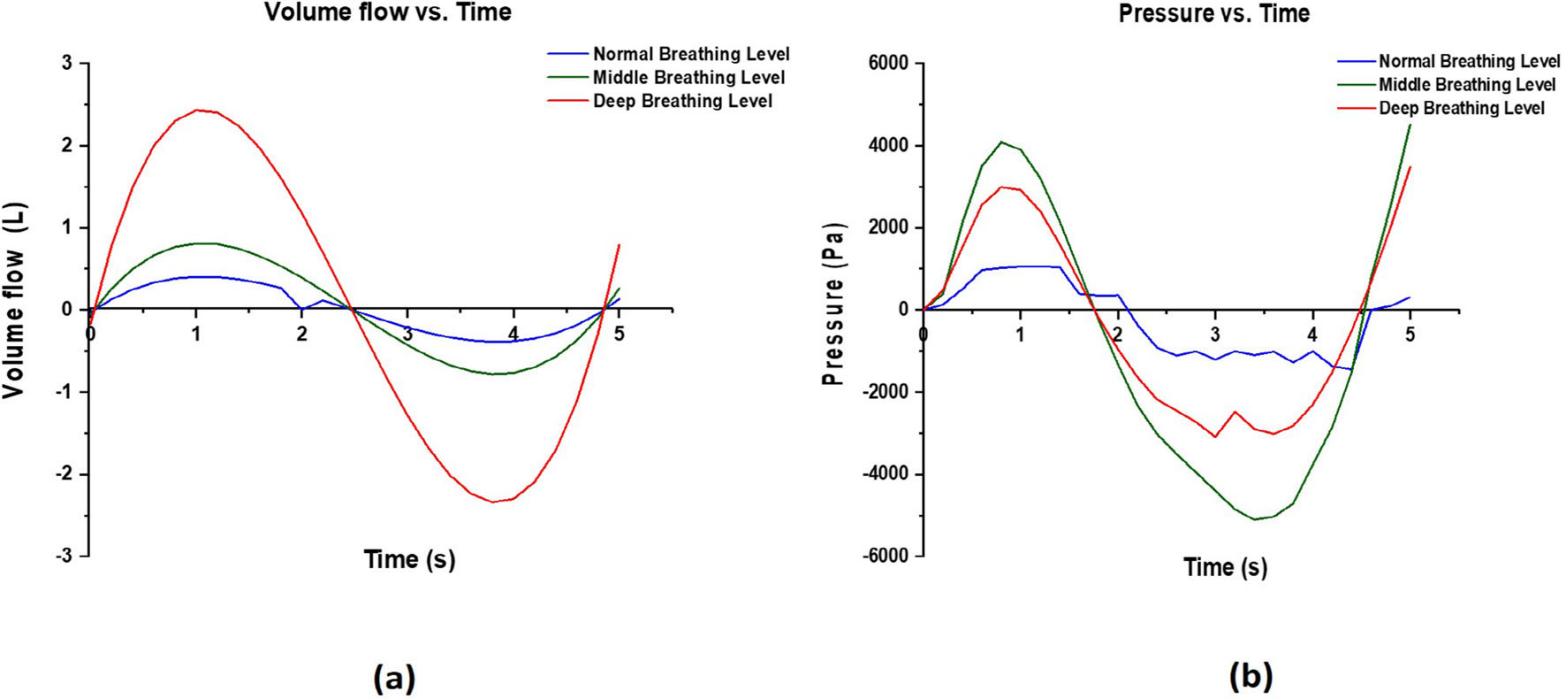
The relationship between **a** volumetric flow and time and **b** pressure and time for the normal, middle, and deep breathing conditions

Figure 5a presents the relationship between the volume flow and time for normal, middle, and deep breathing conditions. The curve of volumetric flow increases gradually until exhalation, reaching a maximum value of 0.5 L/s, and then goes down until the end of exhalation. Volumetric flow has negative values during inhalation because of the change of air direction; the volume flow rate increases with the increase in the difficulty of breathing. The normal state achieved 0.5 L/s as the highest value, whereas the corresponding middle and deep breathing values were 0.7 L/s and 2.3 L/s, respectively. Therefore, velocity increases with the breathing effort and the corresponding volume flow rate increases to fill the lungs.

The pressure during the breathing cycle at the inlet of the nostrils is illustrated in Fig. 5b and follows the same trend as the velocity profile. The pressure increased with the change of breathing condition from normal to deep. The maximum pressure occurred near 0.9 s at exhalation (about 4000 Pa), whereas the maximum pressure at inhalation occurred at 3.5 s achieving a value of − 5000 Pa.

### The distribution of velocity versus time

**Fig. 6:**
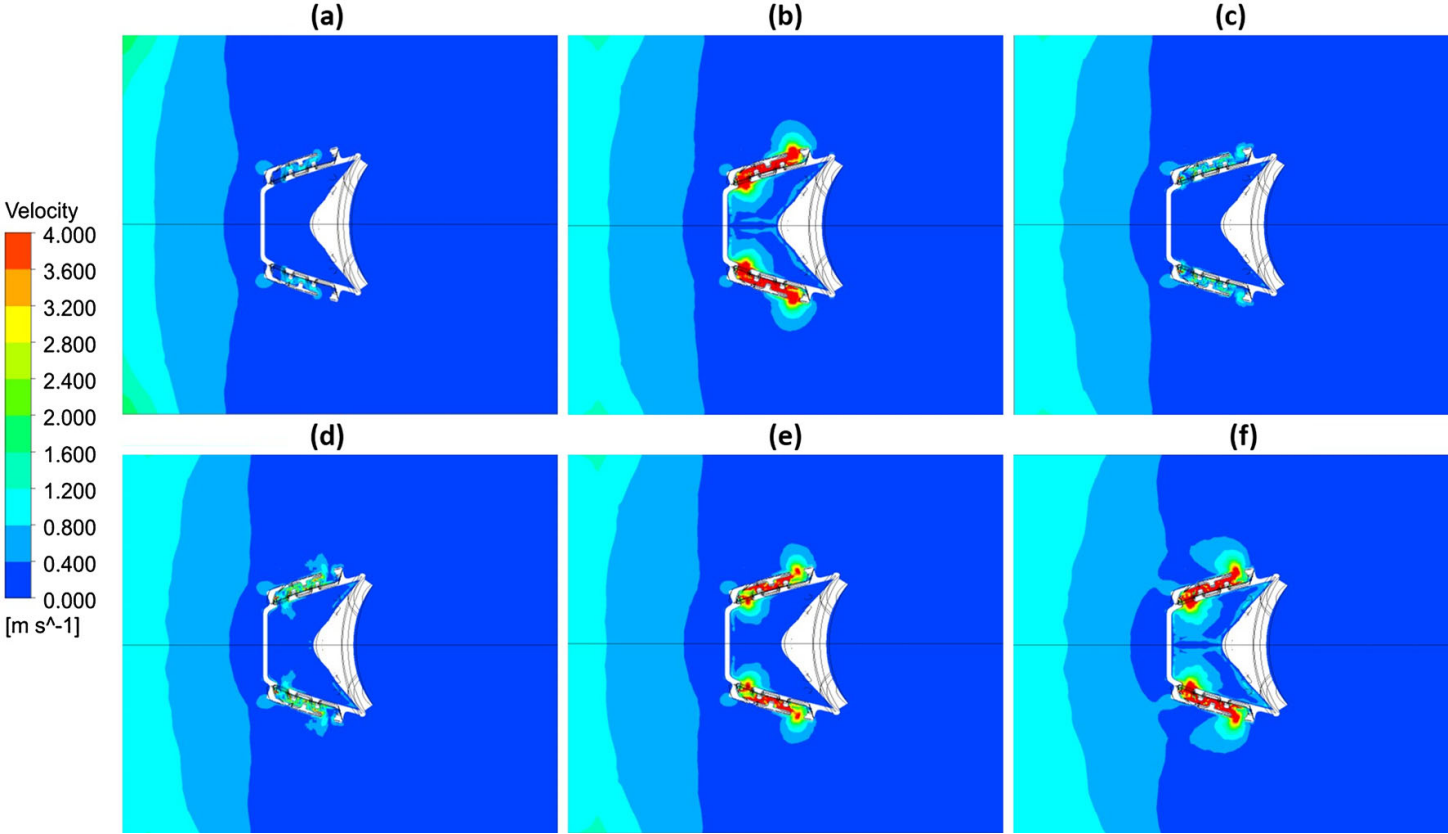
Velocity distribution for the top section of the mask at times a = 0, b = 0.4 s, c = 1.0 s, d = 2.6 s, e = 3.8, and f = 5.0 s

As an example, the velocity of the air and included virus particle distribution in a mask cross-section (plan view) for deep breathing is presented in Fig. 6. It shows the speed for the inner, outer, and filter domains during exhalation (Fig. 6a, b and c). For inhalation, results are shown in Fig. 6d, e, and f. Because of the square wave shape of the filter area, increased velocity inside the filter was observed. The velocity achieved the highest value at half of exhalation and inhalation of the breathing cycle, and the speed of the mixture varies from 4 ms^−1^ to zero at 2.6 s. The turbulence flow level increased at the cross section of the filter because the high air velocity in the form of swirls giving rise to the centrifugal force at the air vortex, which makes the air pass many times through the filter.

**Fig. 7:**
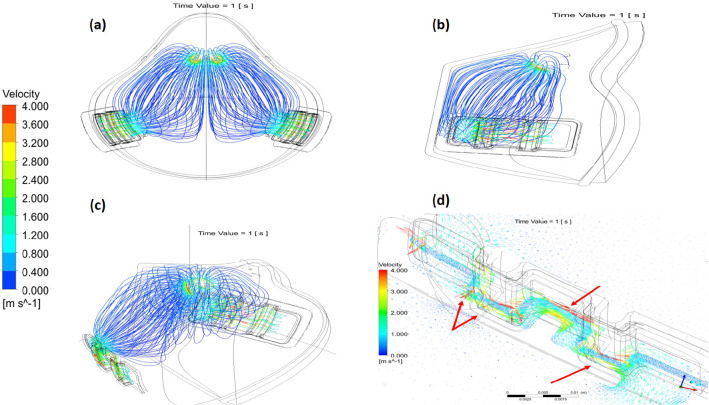
Velocity streamlines during normal breathing at the inner and filter domains of the mask at 1 s, where (a), (b), (c) are different views of the mask and (d) demonstrates the airflow streamlines of the inner domain of the filter

Figure 7 shows the streamlines of the velocity distribution across the filter and in the inner domain at 1 s. The results show that there is no resistance to the ventilated air inside the mask that leads to more comfortable conditions and easy breathing. It is clearly shown the maximum airspeed occurred in the filter media due to the square-waveform shape; therefore, the cross section area decreased, which caused turbulent flow. Therefore, the airflow forced virus particles to hit the filter walls.

Figure 7d takes a closer look at the velocity vectors inside the filter during exhalation at 1 s. It clearly shows that velocity vortex directions dramatically changed in the filter domain. As a result, the velocity increased beside the filter wall preventing the virus particles from scaping the filter media to the environment and infect other people.

### The distribution of pressure and temperature versus time

**Fig. 8:**
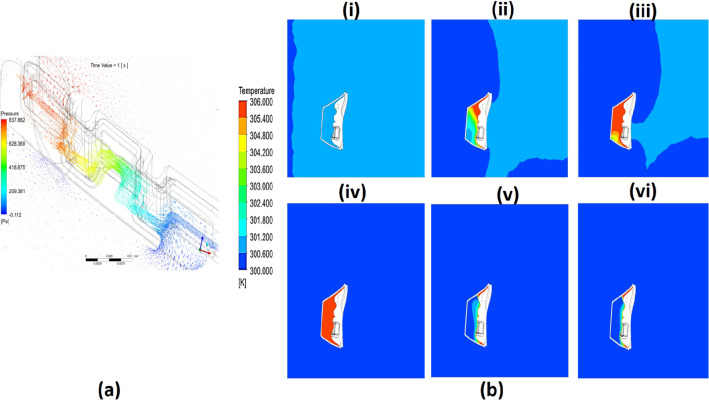
(a) Pressure distribution streamlines for filter domain. (b) Temperature distribution at cross top section of the full domain for (i) t = 0 s, (ii) t = 0.4 s, (iii) t = 1.0 s, (iv) t = 2.6 s, (v) t = 3.8, and (vi) t = 5.0 s

Figure 8a shows the pressure distribution across the filter during exhalation at 1 s where the inner mask domain undergoes the high pressure; the pressure decreases across the filter until it reaches almost the atmospheric condition. The reason for this phenomenon is that porous filter material prevents infectious virous particles from passing through the filter and sustains the pressure produced because of mask wall friction.

Figure 8b shows the temperature distribution for the cross section of the mask at different times. It shows that the cheek surfaces and air exhalation are the sources of heat, where the temperature increases from 300 to 306 K. Figure 8b shows the temperature during the exhalation process. The air that passes from the nostrils heats the interior domain until the temperature reaches the maximum values. During inhalation of the breathing cycle, only cheek surfaces heat the inner volume of air inside the mask where smaller thicknesses beside the surface show a high temperature, and the full domain remains at ambient temperature. Inhaled air became a source of cooling in this case.

## Conclusions

A novel transparent reusable face respirator which can be economically and easily produced worldwide has been created. Several mask prototypes were manufactured using additive manufacturing 3D printing to examine the design and satisfaction while wearing it. The masks were then made using silicone mold dies with clear epoxy. There are four points for fixing the mask over the face from top to bottom of the mask allowing the sealant to prevent any leakage. Filter media plays a crucial role in this study due to the square-waveform shaped design of the filter area, which provides significant turbulent flow to force any infectious virus particles to hit the filter wall. Pressure, velocity, and temperature distributions during three different breathing levels of normal, intermediate, and deep show clearly that the mask is comfortable to wear and the amount of oxygen entering the mask is sufficient while it performs the crucial task of virous particle filtration. Further work is in progress to improve the design, flow modeling and manufacture of the mask.

## Data Availability

All data of this paper are contained in the manuscript.
